# Psychosocial morbidity profile in a community based sample of low back pain patients

**DOI:** 10.1038/s41598-021-82324-y

**Published:** 2021-01-28

**Authors:** Mir Mahmood Asrar, Babita Ghai, Dhanuk Pushpendra, Dipika Bansal

**Affiliations:** 1 Clinical Research Unit, Department of Pharmacy Practice, National Institute of Pharmaceutical Education and Research, SAS Nagar, Mohali, Punjab 160062 India; 2 Department of Anesthesia, Postgraduate Institute of Medical Education and Research, Chandigarh, 160012 India

**Keywords:** Health care, Risk factors, Signs and symptoms

## Abstract

Low back pain (LBP) is a major health concern and is closely associated with psychosocial morbidity and diminished Health-related quality of life (HRQoL). This is minimally investigated in community-based samples of developing nations like India. This study is aimed to specifically investigate the exposure-outcome associations between LBP and burden of disability (Modified Oswestry questionnaire (MODQ)), psychological morbidities *(*Depression, Anxiety and Stress Scale (DASS-21))*,* and HRQoL *(*Short Form -12 version 2 (SF12V2)*.* A Cross-sectional study using a community-based sample of LBP positive population was conducted. The range of treatment options sought was also collected. Chi-square tests and independent t-test were used to analyze the data. Of 1531 recruited participants, 871(57%) were identified as LBP positive of whom 60% were females. Mean (SD) of age and pain intensity of LBP patients was 33 (11) years and numeric rating scale4.2 (2.6) respectively. Two-third reported minimal/moderate disability. Mean (SD) scores of depression 11.87 (4.05), anxiety (8.32), stress 13.7 (5.98), physical and mental summary scores of SF-12v2 were 47.9 (7.4) and 42.2 (10.4). A multitude of remedial options was sought for the ailment. LBP causes significant disability and psychological morbidity among affected population. This may adversely affect their HRQoL and subsequently productivity. Acupuncture was a preferred treatment sought by Indian LBP patients.

## Introduction

Despite extensive global research efforts, low back pain (LBP) remains a troublesome issue both for physicians and patients due to substantial cost burden and fewer recovery rates. Global Burden of Disease Study 2016 (GBD 2016) reported LBP as the leading cause for years lived with disability (YLDs) contributing 57.6 million YLDs^1^.

The specific etiology remains uncertain, often labeling it to be non-specific. It is often associated with sedentary life style, obesity, smoking and physically demanding jobs such as heavy weight lifting, physical and mental comorbidities^2^. Though, LBP affects all age groups, disability related to it is predominant in working population and most troublesome in low and middle-income countries where it has risen by more than 50% since 1990^3,4^.

LBP affects the physical, emotional and social functioning of the affected individual. The imposed biophysical limitations impair physical functioning and adversely affect the general health and reconditioning (weight gain and loss of muscle tone)^5^. The adverse psychological and social impact on patient’s health leads to clinically diagnosed or self-reported psychological morbidities^6,7^. This psychological disrupt is manifested as irritability, insomnia, depression, anxiety and somatic complaints^8,9^. This all impairs the societal participation, function and financial aspect of the affected individual leading to poor quality of life (QoL)^10,11^. Further, the cost of LBP is listed high as compared to other disorders, and the direct medical costs are even higher when associated with psychosocial impact, involving elaborate diagnostic procedures and treatment essentially employing the use of tertiary pain clinics and multi-professional teams^12,13^.

The present report is the second part of the large cohort study^3^ with an aim to determine the burden of disability, psychological morbidities, and QoL in participants diagnosed with LBP as well as the range of treatment options sought for LBP. This study provides a better insight of the burden and presentation of psychological features and their treatment seeking behavior within LBP population recruited from community in the context of a developing nation like India.

## Study methods

### Study design and settings

The original study is a large community based cross-sectional study using stratified multi-level systematic random sampling and a structured data collection form (DCF). The participant enrolment happened during January 2015-Dec 2016. It was conducted in Punjab, Haryana and Chandigarh area of northern India^3^. This project was registered in Clinical trial registry of India vide registration number CTRI/2014/11/005158 dated on 03/11/2014 (http://ctri.nic.in/Clinicaltrials/pmaindet2.php?trialid=8968&EncHid=&userName =).

The present report is the second in line of the original large cohort study. The patients diagnosed with LBP in the initial survey were further interviewed for a detailed evaluation of functional disability, psychological morbidities and QoL using standardized questionnaires. Further, the range of treatment options sought for LBP. The Strengthening of Reporting of Observational Studies in Epidemiology (STROBE) criteria was followed while reporting this study^14^. The study was approved by Institute Ethics Committee (IEC), PGIMER, Chandigarh vide approval number PGI/IEC/2014/2 dated March 11, 2014. The study was conducted in accordance with the guidelines and regulations proposed by the IEC, PGIMER Chandigarh.

### Participants

Adult participants of either gender between 18 and 65 years of age diagnosed with LBP in initial interview, willing to participate in further detailed study were interviewed^3^. The study followed the definition proposed by the International Association for the Study of Pain (IASP) to define LBP and face-to-face interview was used to determine study eligibility. Subjects not willing to participate were excluded from the study. The purpose and procedure of the study was thoroughly explained while obtaining written informed consent (IC) from all the prospective participants.

### Data collection

The information compromising socio-demographic details and medical co-morbidities were obtained from the initial DCF. A trained investigator having good linguistic competence (in English, Punjabi and Hindi) comprehensive knowledge of disease and competent in conducting survey and was employed for data collection.

### Study outcomes assessment tools

The outcomes assessed were functional disability using Modified Oswestry Low Back Pain Disability Questionnaire (MODQ)^15^, psychological morbidity using Depression, Anxiety and Stress Scale (DASS-21)^16^, quality of life (QoL) using Short Form -12 version 2 (SF12V2)^17^, and the range of treatment options sought for LBP using DCF. MODQ is a validated tool in Indian settings and very specific to the LBP population for assessing functional disability. The research team is well versed in using MODQ in their previous studies too. DASS 21 contains all the aspects of psychological morbidity and is a reliable and valid measure of depression, anxiety, and stress in the clinical and non-clinical adult population and different cultural and ethnic groups in a composite manner. Since this was a community setting, we required a concise questionnaire incorporating all the psychosocial aspects. SF-12v2 is the shortened form of SF36v2, which is employed for the generic assessment of the health-related quality of life. The inclusion of SF12 was deemed to its validity and easy to administer and co-administration with other disease-specific instruments.

### Modified Oswestry low back pain disability questionnaire (MODQ)

The questionaire consists of ten items about the pain intensity, lifting, ability to walk, ability to care for himself, ability to sit, ability to stand, sexual function, social life, ability to travel, quality of sleep. For every question asked has six different options which describe the different potential plots in the subject’s experience concerning the topic^15^. The participants responded with the statement, which mostly resembled with their present situation. Each item scored from zero to five, and the higher values indicate a more significant disability. The scores obtained are transformed into percentages, zero indicating no impairment, and 100 is the maximum disability possible.

### Depression, anxiety and stress scale (DASS-21)

The Depression, Anxiety, and Stress Scale-21 Items (DASS-21) consist of three self-report scales intended to measure the emotional states of depression, anxiety, and stress. Each scale of DASS-21 contains seven items. The depression scale assesses hopelessness, dysphoria, self-deprecation, devaluation of life, lack of interest/involvement, anhedonia, and inertia. The anxiety scale assesses skeletal muscle effects, autonomic arousal, situational anxiety, and subjective experience of anxious affect. It assesses difficulty relaxing, nervous arousal, and being easily upset/agitated, irritable/over-reactive, and impatient. The stress scale is sensitive to levels of chronic nonspecific arousal. The Scores for each scale are calculated by summing the scores for the relevant items^16^.

### Short Form-12 version 2 (SF-12V2)

The SF-12v2 instrument consisting of twelve questions to evaluate the functional health and well-being from the patient’s perspective was employed^17^. The SF-12v2 is a useful, valid and reliable measure of physical and mental health which covers 8 health domains viz Physical Functioning, General Health, Role Physical, Bodily Pain, Social Functioning, Vitality, Mental Health and Role Emotional and domains. The SF 12v2 scales are scored using Likert’s method with raw scores being summated and linearly transformed into 0–100 scale (100 indicate the best level and 0 the worst). Thus, higher scores represent better health status.

### Treatment seeking for LBP

Range of treatment options sought for LBP such as allopathy, Ayurveda, homeopathy, complementary and alternative medicine (CAM) were recorded.

### Statistical analysis

Data were recorded in Microsoft Excel and transported to SPSS version 22 for analysis. The prevalence of functional disability and psychological morbidities; depression, anxiety, and stress was presented as a proportion of the sample with a 95% confidence interval (95% CI.). Chi-square and independent t-test were applied to examine categorical and quantitative variables, respectively. Throughout the analysis probability value (*p* value) < 0.05 was considered as statistically significant.

### Ethics approval and informed consent

Clearance was obtained from the ethics committee of PGIMER, Chandigarh. Written informed consent was also obtained from the patients enrolled in the study. All data has been kept confidential.

## Results

**Figure 1:**
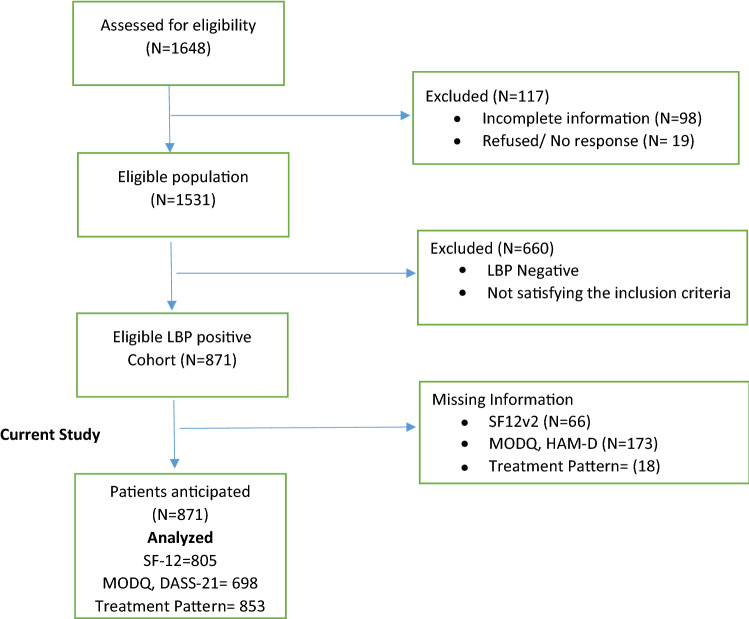
Flowchart of study sample selection.

A total of 1531 participants were enrolled in the primary study, amongst 871 (57%) participants were identified positive for LBP. Of these potentially eligible 871 participants, 805 (92%) consented to participate for further detailed psychosocial evaluation (Fig. 1). The response rate of the participants for enrollment in the study was SF12 (92%), MODQ (80%), DASS 21 (80%) and treatment pattern (98%).

### Sociodemographic and clinical characteristics

The mean (SD) age of total cohort (n = 1531) was 32.0 (10) years, with significantly higher age 33 (11) years in LBP positive patients (*p* < 0.05). The mean (SD) body mass index (BMI) was 22.6 (4.5) which were comparable in LBP positive and negative participants. Nearly, 50% of the participants were eligible for medical reimbursement with significantly higher proportion 474 (54%) in LBP positive subjects (*p* < 0.05).

Among LBP positive participants (n = 871), majority 594 (68%) belongs to younger (18–35 year) age group. The mean (SD) BMI was 22.5 (4.4) kg/m^2^ with only one-fourth being over-weight and majority 532 (61%) amongst LBP positive patients having healthy BMI. Females had lower BMI 21.5 (4.2) kg/m^2^ as compared to males 23.9 (4.4) kg/m^2^, *p* < 0.05. The overall mean (SD) pain intensity was reported to be moderate; numeric rating scale 0-to-10 (NRS 0–10) score 4.2 (2.6) with females reporting significantly lower pain intensity [4.1 (2.6)] as compared to males 4.5 (2.5), *p* < 0.05. Majority had a history of the blue-collar job 719 (83%) and nearly half had regularly practiced load lifting activity (Table 1).

**Table 1:** Baseline sociodemographic characteristics.

Characteristics	Total N = 1531	LBP + 871 (57%)	LBP− 661 (43%)
Age, years, M (SD)	32 (10)	33 (11)	31 (9)
BMI, kg/m^2^, M (SD)	22.6 (4.5)	22.5 (4.4)	22.8 (4.6)
**Medical reimbursement, N (%)**			
Yes	755 (49)	471 (54)	284 (43)
No	776 (51)	399 (46)	377 (57)

Characteristics	Total 871 (100)	Male N = 346	Female N = 525
Age, years, M (SD)	33 (11)	34 (10)	32 (11)
**Age group, years**			
18–35	594 (68)	230 (67)	364 (69)
36–60	263 (30)	108 (32)	155 (30)
> 60	14 (2)	8 (2)	6 (1)
Height, cm, M (SD)	166 (9)	168 (9)	164 (8)
Weight, kg, M (SD)	62 (12)	67 (12)	58 (11)
BMI, kg/m^2^, M (SD)	22.5 (4.4)	23.9 (4.4)	21.5 (4.2)
**BMI range, kg/m**^**2**^			
< 18.50	130 (15)	27 (8)	103 (20)
18.50–24.99	532 (61)	196 (57)	336 (64)
> 25	208 (24)	123 (35)	85 (16)
Pain intensity, M (SD)	4.2 (2.6)	4.5 (2.5)	4.1 (2.6)
**Medical reimbursement**			
Yes	472 (54)	191 (55)	281 (53)
No	399 (46)	155 (45)	244 (47)
**Type of Job**			
White color	152 (17)	64 (19)	88 (11)
Blue color	719 (83)	282 (81)	437 (83)
**Type of activity**			
Standing/sitting	426 (49)	169 (49)	257 (49)
Walking/lifting	445 (51)	177 (51)	268 (51)

M (SD): mean (standard deviation), N (%): number (percentage), BMI: body mass index.

Data represented as mean (standard deviation) unless otherwise specified N (%). *p < 0.05.

### Outcome measures

#### Functional disability (n = 698)

478/698 (68%) subjects had reported minimal/moderate functional disability and 220/698 (32%) suffer with severe, crippled or bed bound disability at one-year follow-up. Significantly higher proportion of females 139/395 (35%) had severe disability as compared to males 81/303 (27%), *p* < 0.05. The overall mean (SD) MODQ score was 17.54 (7.3), with significantly higher scores in females 18.1 (7.3) as compared to males 16.8 (7.2) (*p* < 0.05) (Table 2).

**Table 2:** Functional disability (N = 698).

Disability	Total N = 698 N (%)	Male N = 303 N (%)	Female N = 395 N (%)	*p* value
Minimal/Moderate disability	478 (68)	222 (73)	256 (65)	< 0.05
Severe disability crippled/bed bound	220 (32)	81 (27)	139 (35)	
MODQ Score M (SD)	17.5 (7.3)	16.8 (7.2)	18.1(7.3)	< 0.05

N: Number of Subjects; %: Percentage.

#### DASS21 (n = 698)

##### Depression

Majority of the patients reported DASS-21 scores within mild range 276/698(40%), having mean (SD) DASS-21 scores for depression scale 11.87 (4.05) with significant difference among males and females (*p* < 0.05). 226/698 (32%) subjects suffered with moderate depression and 13/698 (2%) with severe depression. A significantly higher proportion of females suffer with mild depression 174/395 (44%) as compared to males 102/303 (34%) (*p* < 0.05) (Table 3).

**Table 3:** Depression, anxiety and stress characteristics of subjects with LBP (N = 698).

Depression	Total N = 698	Male N = 303	Female N = 395	*p* value
Normal	183 (26)	94 (31)	89 (23)	< 0.05
Mild	276 (40)	102 (34)	174 (44)	< 0.05
Moderate	226 (32)	99 (33)	127 (32)	0.88
Severe	13 (2)	8 (3)	5 (1)	0.18
Score M(SD)	11.87 (4.05)			
**Anxiety**				
Normal	302 (43)	158 (52)	144 (37)	< 0.05
Mild	126 (18)	48 (16)	78 (20)	0.18
Moderate	206 (30)	70 (23)	136 (34)	< 0.05
Severe	59 (9)	26 (9)	33 (8)	0.92
Extremely severe	5 (1)	1 (0.3)	4 (1)	0.29
Score M(SD)	8.32 (4.48)			
**Stress**				
Normal	378 (54)	168 (55)	210 (53)	0.55
Mild	172 (25)	78 (26)	94 (24)	0.55
Moderate	131 (19)	46 (15)	85 (22)	< 0.05
Severe	17 (2)	11 (4)	6 (2)	0.78
Score M(SD)	13.77 (5.98)			

N: number of subjects under study; Data presented as number (percentage); M: Mean; SD: Standard Deviation.

##### Anxiety

The mean (SD) anxiety score on DASS-21 was reported to be 8.32 (4.48). 126/698 (18%) patients reported mild, 206/698 (30%) moderate, and 59/698 (9%) reported severe anxiety, respectively. A significantly higher proportion of females suffered with moderate anxiety 136/395 (34%) as compared to males 70/303 (23%) (*p* < 0.05) (Table 3).

##### Stress

The mean (SD) stress score on DASS-21 scale was reported to be 13.7 (5.98). 172/698 (25%) patients reported mild, 131/698 (19%) reported moderate, and 17/698 (2%) reported severe stress (Table 3). A significantly higher proportion of females suffer with mild anxiety 85/395 (22%) as compared to males 46/303 (15%) (*p* < 0.05).

### Health related quality of life

The mean (SD) of physical and mental summary scores of SF-12v2 were 47.9 (7.4) and 42.2 (10.4), respectively. Among various domains, the mean (SD) highest and lowest reported scores were in Vitality 52.7 (10.1) and Role emotional 38.8 (17.6) domains, respectively (Table 4). Further, subgroup analysis reported that gender, age, BMI, type of job and type of activity do not influence the physical and mental summary scores (Table 5).

**Table 4:** SF-12v2 summary scores.

SF-12 Domains	Total N = 805 M (SD)	Male N = 336 M (SD)	Female N = 469 M (SD)	*p* value
Physical functioning	46.3 (9.0)	46.7 (8.8)	46.1 (9.1)	0.39
Role physical	45.0 (13.7)	45.2 (13.4)	44.9 (13.9)	0.73
Bodily pain	44.5 (8.7)	44.4 (8.7)	44.5 (8.6)	0.79
General health	47.2 (8.7)	47.2 (8.8)	47.1 (8.6)	0.84
Vitality	52.7 (10.1)	52.6 (10)	52.6 (9.0)	0.91
Social functioning	41.0 (9.2)	41.1 (9.1)	41.0 (9.3)	0.92
Role emotional	38.8 (17.6)	39.0 (17.5)	38.8 (17.7)	0.92
Mental health	43.5 (9.9)	43.3 (10.2)	43.5 (9.6)	0.84
Physical component summary	47.9 (7.4)	48.1 (7.5)	47.8 (7.3)	0.53
Mental component summary	42.2 (10.3)	42.1 (10.4)	42.3 (10.2)	0.82

M: Mean; SD: Standard Deviation.

**Table 5:** Scores of various domains in various subgroups (N = 805).

	PCS M (SD)	*p* value	MCS M (SD)	*p* value
**Age category**				
< 35 Years	42.7 (9.9)	0.91	48.0 (7.0)	0.13
> 35 Years	41.5 (11.0)		47.9 (7.8)	
**BMI**				
Underweight	46.9 (7.5)	0.23	41.6 (9.5)	0.34
Normal	48.0 (7.2)		42.7 (10.9)	
Overweight	48.8 (7.6)		41.6 (9.3)	
**Type of job**				
White collar	48.9 (7.5)	0.08	42.7 (10.4)	0.53
Blue collar	47.7 (7.3)		42.1 (10.3)	
**Type of activity**				
Standing/sitting	47.9 (7.3)	0.90	42.6 (10.5)	0.36
Lifting/walking	47.9 (7.4)		41.9 (10.1)	
**Medical reimbursement**				
Yes	48.0 (7.3)	0.57	42.1 (10.7)	0.80
No	47.7 (7.4)		42.4 (9.8)	

BMI: Body Mass Index; PCS: Physical Component Score; MCS: Mental Component Score; M: Mean; SD: Standard Deviation.

### Treatment seeking (n = 853)

Patients sought a range of treatment options. The most preferred treatments were acupressure 333 (39%), medical doctor/private hospital/public hospital 140 (16%), Ayurveda 116 (14%), and physiotherapy 66 (8%), homeopathy 37 (4%), chiropractic 29 (2%), pharmacy 16 (2%). Significantly higher proportion of males preferred acupressure 150/339 (44%) relative to females 183 (36%) *p* < 0.05 (Table 6).

**Table 6:** Treatment received by subjects with low back pain (N = 853).

Treatment	Total N = 853 N (%)	Male N = 339 N (%)	Female N = 514 N (%)	*p* value
Acupressure	333 (39)	150 (44)	183 (36)	< 0.05
Medical doctor/ Private hospital/ Public hospital	140 (16)	56 (17)	84 (16)	0.95
Ayurveda	116 (14)	48 (14)	68 (13)	0.70
Physiotherapy	66 (8)	18 (5)	48 (9)	< 0.05
Homeopathy	37 (4)	19 (6)	18 (4)	0.14
Chiropractic	29 (3)	11 (3)	18 (4)	0.84
Pharmacy	16 (2)	6 (2)	10 (2)	0.85
Other	116 (14)	31 (9)	85 (16)	< 0.01

N: Number of Subjects; %: Percentage.

## Discussion

The present report describes psychosocial profile of patients suffering from LBP enrolled in a large community based study reporting a high prevalence of 57% in the initially enrolled subject cohort of 1531^3^. These LBP positive subjects were further interviewed to assess the psychosocial morbidity, disability and quality of life using DASS-21, MODQ and SF-12V2 questionnaires, respectively. The range of treatment options sought for LBP was also documented.

Two-third subjects reported to suffer from mild to moderate functional disability while one–third suffered from severe to crippled disability. Severe disability was highest in working age groups (18–35 years). LBP is found to have a considerable impact on work productivity as affected participants reported on average six days of work absenteeism in previous month due to paint. This is especially concerning in a country like India where informal employment is usually the rule and possibilities for job switching are almost nadir. Further, it needs to be emphasized that occupational musculoskeletal health policies, such as regulatory framework for heavy physical work are often either non-existing or poorly monitored^18^. As predicted earlier, GBD 2016 reported remarkable rise in the burden LBP especially in Low and Middle income countries with LBP contributing 57.6 million (40.8–75.9 million) YLDs, largely because of aging and rapidly expanding global population^1,2^. The affected subjects reported to have difficulties in performing their activities of daily living resulting in greater isolation, less motivation and finally leading to functional disability of various grades^19^.

The disabilities associated with LBP may be an interplay of psychological factors and altered bodily functions^20^.Though severe depression was reported in 2% participants, two-third subjects did report some grade of depressive symptoms in the present study. Affected subjects are unable to fulfill their traditional and expected social roles in a society^21^. The deliberation of psychological factors concerning LBP is highly relevant. The negative psychological attributes such as pain Catastrophizing and pain-related fear avoidance behavior, increased somatic awareness and depression are associated with higher perceptions of pain and disability^22^. Gatchel et al. proposed an apparent worsening of psychosocial sequelae in patients with chronic relative to acute pain^23^.

More than half of the affected subjects reported anxiety in our study. This is supported by an earlier study from Tehran enrolling 112 patients with chronic LBP, 46.4% of patients reported symptoms of anxiety and 48.2% patients had depressive disorder (27). Further, another study from Portugal enrolling 284 subjects with chronic LBP reported 51.4% had both the symptoms of anxiety and depression, 21.5% had symptoms of anxiety alone and 6.7% had depressive symptoms while having their first consultation in multidisciplinary pain clinic. The study had used Hospital Anxiety and Depression Scale to measure the psychological morbidity^24,25^. These observations ignite the requirement screening of anxiety and depression before treatment as independent symptoms in patients with LBP to design more tailored and effective multidisciplinary treatments. The imposed physical limitations result in psychological distress, anxiety and influence the coping strategies and course of pain. Chronic musculoskeletal pain symptoms have enormous economic and subsistence consequences as these usually lead to a loss of independence and social identity.

Half of the affected subjects reported some sort of stress in the study cohort. Tsuboi Y et al. reported higher perceived stress to be significantly linked with a higher incidence of memorable LBP in the prior month in 571 eldercare workers^26^. Mindfulness-Based Stress Reduction is extensively adopted and listedto produce definite impacts on psychological well-being and symptoms of many disorders including LBP^27^.

Overall LBP had significant adverse impact on QoL of study participants. LBP affects all life realms from moderately basic self-care activities to advance social interactions, leisure activities, work, and ultimately have a profound impact on the QoL^28^. In a large cohort of 1502 patients endeavoring for multidisciplinary spine care, the individual and societal impact of LBP is very high. Specifically, QoL and workability are poor and health care costs are twice as high corresponded to patients seeking primary LBP care^29^. However, Pellisé et al. reported that LBP possesses a little effect on HRQoL in adolescents^30^. Ludwig et al. reported that LBP-related activity limitations had little influence on both self-rated overall health^31^. Choi et al. reported the negative QoL in the Korean population^32^.

### Treatment seeking behavior

Management of LBP was dominated by acupressure (39%) followed by Medical doctor/ Private hospital/ Public hospital (16%), Ayurveda (14%), Physiotherapy (8%) and Homeopathy (4%) in present report. The preference for acupressure may be related to the belief and cultural practices in India. Acupressure is believed to be originated from India or China and is based on Indian ancient philosophy. In Indian culture it is usually believed to be non-invasive and without side effects. It is believed to be inexpensive and without side effects. Acupressure has been reported to provide better outcome compared to usual care^33,34^.

A large proportion of the participants preferred Ayurveda (14%) in the present study. Ayurveda remain the most ancient practice yet living tradition in India and rising interest of traditional medicine globally^35^. Herbal drugs in Indian society are widely accepted to improve one’s condition with fewer side effects, which may be the reason for increased use of the complementary and alternative medicine both in the developing and developed countries^36^. However, efforts are required to introduce and validate pharmacoepidemiological evidence concerning the safety and practice of Ayurveda medicines^37^. Various treatment options are available for CLBP management. However, there is insufficient or conflicting evidence about these modalities^38^.

16% sought medical help by visiting medical practitioner public or private. Pharmacological methods seem inadequate as a sole treatment to CLBP^38^. In LBP, limited evidence is available to guide health care professionals on advanced use of recommended evidence-based practice^39^. Probably alternative and complementary medicine is usually tried as initial treatment modalities because of perception of low/no side effect as reflected by our results.

The strength of the study lies in the efficiency of the data acquisition with regards to exposure. To our best knowledge, our study is the first to determine the psychological morbidity, disability and quality of life in patients with LBP in Indian population. However, the limitations was the limited sample size and no follow up. Further, we understand the predictive limitations of cross-sectional studies, i.e. the evidence of a temporal relationship between LBP and psychosocial morbidity can’t be ascertained in our data. Future efforts should assess psychosocial morbidity and functional disability in larger cohorts in a longitudinal manner to fully understand the relationships between psychosocial morbidity, functional disability, and HRQoL in LBP. The current findings may be generalized qualitative and quantitatively to other developing countries, though caution is recommended given the limited sample size or when generalizing the current findings to participants suffering comorbidity.

## Conclusion

To our best knowledge, this is the largest study of LBP evaluating psychological morbidity, disability, and Health-Related Quality of Life in an Indian population. LBP results in significant disability, which critically affects the quality of life of the patients. A quarter of patients reported depression. Extensive treatment coping strategies were adopted by the patients with more preference given to acupuncture.

## References

[1] Vos T (2017). Global, regional, and national incidence, prevalence, and years lived with disability for 328 diseases and injuries for 195 countries, 1990–2016: a systematic analysis for the Global Burden of Disease Study 2016. Lancet (London, England).

[2] Hartvigsen J (2018). What low back pain is and why we need to pay attention. Lancet (London, England).

[3] Bansal D, Asrar MM, Ghai B, Pushpendra D (2020). Prevalence and impact of low back pain in a community-based population in northern India. Pain Phys..

[4] Gurdan Z (2017). Effect of pre-drilling on intraosseous temperature during self-drilling mini-implant placement in a porcine mandible model. J. Oral Sci..

[5] Clark S, Horton R (2018). Low back pain: a major global challenge. Lancet (London, England).

[6] Bair MJ, Robinson RL, Katon W, Kroenke K (2003). Depression and pain comorbidity: a literature review. Arch. Intern. Med..

[7] Baumeister H, Knecht A, Hutter N (2012). Direct and indirect costs in persons with chronic back pain and comorbid mental disorders—a systematic review. J. Psychosom. Res..

[8] Kishore J, Reddaiah VP, Kapoor V, Gill JS (1996). Characteristics of mental morbidity in a rural primary heath centre of haryana. Indian J. Psychiatry.

[9] Claiborne N, Vandenburgh H, Krause TM, Leung P (2002). Measuring quality of life changes in individuals with chronic low back conditions: a back education program evaluation. Eval. Program. Plan..

[10] Quality of Life Measures. in *Handbook of Disease Burdens and Quality of Life Measures* 4304-4304 (Springer New York, 2010).

[11] Darzi MT, Pourhadi S, Hosseinzadeh S, Ahmadi MH, Dadian M (2014). Comparison of quality of life in low back pain patients and healthy subjects by using WHOQOL-BREF. J. Back Musculoskelet. Rehabil..

[12] Index. *Best Practice & Research Clinical Rheumatology* **16**, I1–I2 (2002).

[13] Patrick DL, Erickson P (1996). Health Status and Health Policy: Quality of Life in Health Care Evaluation and Resource Allocation.

[14] von Elm E (2007). Strengthening the Reporting of Observational Studies in Epidemiology (STROBE) statement: guidelines for reporting observational studies. BMJ.

[15] Fairbank JC, Couper J, Davies JB, O'Brien JP (1980). The Oswestry low back pain disability questionnaire. Physiotherapy.

[16] Lovibond, S.H. & Lovibond, P.F. Depression Anxiety Stress Scales. in *PsycTESTS Dataset* (American Psychological Association (APA), 1995).

[17] Ware J, Kosinski M, Keller SD (1996). A 12-Item Short-Form Health Survey: construction of scales and preliminary tests of reliability and validity. Med. Care.

[18] Lucchini RG, London L (2014). Global occupational health: current challenges and the need for urgent action. Ann. Glob. Health.

[19] Marcic M, Mihalj M, Ivica N, Pintaric I, Titlic M (2014). How severe is depression in low back pain patients?. Acta Clin. Croat..

[20] Cai C, Pua YH, Lim KC (2007). Correlates of self-reported disability in patients with low back pain: the role of fear-avoidance beliefs. Ann. Acad. Med. Singap..

[21] Elfering A, Kaser A, Melloh M (2014). Relationship between depressive symptoms and acute low back pain at first medical consultation, three and six weeks of primary care. Psychol. Health Med..

[22] Meyer K, Tschopp A, Sprott H, Mannion AF (2009). Association between catastrophizing and self-rated pain and disability in patients with chronic low back pain. J. Rehabil. Med..

[23] Gatchel RJ, Bernstein D, Stowell AW, Pransky G (2008). Psychosocial differences between high-risk acute vs. chronic low back pain patients. Pain Pract..

[24] Oliveira DS, Velia Ferreira Mendonca L, Sofia Monteiro Sampaio R, Manuel Pereira Dias de Castro-Lopes J, Ribeiro de Azevedo LF (2019). The impact of anxiety and depression on the outcomes of chronic low back pain multidisciplinary pain management-a multicenter prospective cohort study in pain clinics with one-year follow-up. Pain Med. (Malden, Mass.).

[25] Mirzamani SM, Sadidi A, Sahrai J, Besharat MA (2005). Anxiety and depression in patients with lower back pain. Psychol. Rep..

[26] Tsuboi Y, Ueda Y, Naruse F, Ono R (2017). The association between perceived stress and low back pain among eldercare workers in Japan. J. Occup. Environ. Med..

[27] Cherkin DC (2016). Effect of mindfulness-based stress reduction vs cognitive behavioral therapy or usual care on back pain and functional limitations in adults with chronic low back pain: a randomized clinical trial. JAMA.

[28] Montazeri A, Mousavi SJ, Preedy VR, Watson RR (2010). Quality of life and low back pain. Handbook of Disease Burdens and Quality of Life Measures.

[29] Dutmer AL (2019). Personal and societal impact of low back pain: the Groningen spine cohort. Spine.

[30] Pellise F (2009). Prevalence of low back pain and its effect on health-related quality of life in adolescents. Arch. Pediatr. Adolesc. Med..

[31] Ludwig C, Luthy C, Allaz AF, Herrmann FR, Cedraschi C (2018). The impact of low back pain on health-related quality of life in old age: results from a survey of a large sample of Swiss elders living in the community. Eur. Spine J..

[32] Choi YS (2014). How does chronic back pain influence quality of life in koreans: a cross-sectional study. Asian Spine J..

[33] Adams A, Eschman J, Ge W (2017). Acupressure for chronic low back pain: a single system study. J. Phys. Ther. Sci..

[34] Hsieh LL (2006). Treatment of low back pain by acupressure and physical therapy: randomised controlled trial. BMJ.

[35] Patwardhan B, Warude D, Pushpangadan P, Bhatt N (2005). Ayurveda and traditional Chinese medicine: a comparative overview. Evid. Based Complement Alternat. Med..

[36] Ekor M (2014). The growing use of herbal medicines: issues relating to adverse reactions and challenges in monitoring safety. Front. Pharmacol..

[37] Vaidya RA, Vaidya AD, Patwardhan B, Tillu G, Rao Y (2003). Ayurvedic pharmacoepidemiology: a proposed new discipline. J. Assoc. Phys. India.

[38] Day MA (2019). A pilot randomized controlled trial comparing mindfulness meditation, cognitive therapy, and mindfulness-based cognitive therapy for chronic low back pain. Pain medicine (Malden, Mass.).

[39] Hodder RK (2016). Developing implementation science to improve the translation of research to address low back pain: a critical review. Best Pract. Res. Clin. Rheumatol..

